# Recommendations for Abnormal Behaviour Ethograms in Monkey Research

**DOI:** 10.3390/ani11051461

**Published:** 2021-05-19

**Authors:** Andrea Polanco, Brenda McCowan, Lee Niel, David L. Pearl, Georgia Mason

**Affiliations:** 1 Department of Integrated Biology, University of Guelph, Guelph, ON N1G 2W1, Canada; apolanco@uoguelph.ca; 2 Population Health & Reproduction, School of Veterinary Medicine, University of California Davis, Davis, CA 95616, USA; bjmccowan@ucdavis.edu; 3 Department of Population Medicine, University of Guelph, Guelph, ON N1G 2W1, Canada; niell@uoguelph.ca (L.N.); dpearl@uoguelph.ca (D.L.P.)

**Keywords:** welfare, abnormal behaviour, ethogram, convergent validity, management practices, nonhuman primates, rhesus macaque, *Macaca mulatta*

## Abstract

**Simple Summary:**

Monkeys are at risk of performing abnormal behaviours in captivity: signs of poor well-being that are easily recognizable. For practical reasons, researchers typically pool different abnormal behaviours together. However, this is typically performed without assessing whether the behaviours are actually related to each other. Consequently, such subcategories may be misclassifying behaviours. Most importantly, using arbitrary subcategories may reduce their precision to measure animal welfare since different abnormal behaviours are likely to have distinct risk factors and treatments. We therefore investigated the validity of four previously used abnormal behaviour subcategories in laboratory rhesus monkeys (i.e., we assessed whether the subcategories were actually formed of behaviours that co-occurred). These included behaviours previously labelled ‘self-injurious’ (e.g., self-biting), ‘self-stimulating’ (e.g., hair-pulling), ‘postural’ (e.g., floating limb), and ‘motor’ (e.g., pacing). Using a large dataset on 19 different types of abnormal behaviour from over a thousand monkeys, we discovered that none of the subcategories analyzed were valid. Therefore, we reanalyzed the data to create four new valid subcategories that were formed of related behaviours. We additionally identified four behaviours that were not related to any other behaviour and should thus be categorized alone. Once replicated, using this new validated scheme will help researchers and laboratory staff study the conditions that trigger them and develop the most appropriate treatment strategies.

**Abstract:**

Laboratory monkey ethograms currently include subcategories of abnormal behaviours that are based on superficial morphological similarity. Yet, such ethograms may be misclassifying behaviour, with potential welfare implications as different abnormal behaviours are likely to have distinct risk factors and treatments. We therefore investigated the convergent validity of four hypothesized subcategories of abnormal behaviours (‘motor’, e.g., pacing; ‘self-stimulation’, e.g., self-sucking; ‘postural’, e.g., hanging; and ‘self-abuse’, e.g., self-biting). This hypothesis predicts positive relationships between the behaviours within each subcategory. Rhesus macaque (*Macaca mulatta*) data on 19 abnormal behaviours were obtained from indoor-housed animals (*n* = 1183). Logistic regression models, controlling for sex, age, and the number of observations, revealed that only 1/6 ‘motor’ behaviours positively predicted pacing, while 2/3 ‘self-abuse’ behaviours positively predicted self-biting (one-tailed *p*-value < 0.05). Furthermore, ‘self-stimulation’ behaviours did not predict self-sucking, and none of the ‘postural’ behaviours predicted hanging. Thus, none of the subcategories fully met convergent validity. Subsequently, we created four new valid subcategories formed of comorbid behaviours. The first consisted of self-biting, self-hitting, self-injurious behaviour, floating limb, leg-lifting, and self-clasping. The second comprised twirling, bouncing, rocking, swinging, and hanging. The third comprised pacing and head-twisting, while the final subcategory consisted of flipping and eye-poking. Self-sucking, hair-plucking, threat-biting, and withdrawn remained as individual behaviours. We encourage laboratories to replicate the validation of these subcategories first, and for scientists working with other species to validate their ethograms before using them in welfare assessments.

## 1. Introduction

Laboratory monkeys frequently display abnormal behaviours that are rarely seen in the wild, as is the case for many species (see [1]). Such behaviours are more likely to arise from conditions known to cause poor (vs. good) well-being, such as early maternal separation [2], single housing [3,4,5,6,7], indoor housing [5,8,9], and fearful and/or stressful conditions (e.g., exposure to fasting, illumination, intimidation, and space restriction [10]). In rhesus macaques (*Macaca mulatta*), one of the most common species of laboratory monkeys [11,12], at least 19 different abnormal behaviours have been recorded (see Table 1). Pooling these behaviours is common for pragmatic reasons, since recording them separately would be time-consuming and difficult to analyze (e.g., if the data are right-skewed due to many zeros). For instance, prior work has used at least four subcategories ([13]; see Table 1), which include both repetitive stereotypic behaviours (SBs), as well as abnormal stances and postures. SBs are repetitive movements resulting from motivational frustration, brain dysfunction, and/or repeated attempts to cope [14]. Pacing back and forth is the most common form, seen in 78% of single-housed rhesus monkeys [15]. This is followed by other ‘motor’ SBs such as bouncing, rocking, swinging, flipping, spinning, and head-twisting [9,15] (see definitions in Table 1). Other SBs include hair-plucking (i.e., the removal of hair via teeth and/or hands), which affects 8–14% of single-housed laboratory rhesus monkeys [15,16], and self-biting (i.e., when monkeys bite themselves, e.g., on the arm or leg [7]), which affects 14–25% of single-housed rhesus monkeys [15,17]. Other abnormal behaviours are inactive. They include ‘floating limb’ and hunched postures, sometimes referred to as ‘postural’ behaviours (Table 1). ‘Floating limb’ is when a monkey raises their arm(s) or leg(s) without an obvious function, which is seen in 24% of indoor-housed monkeys [18]. A hunched posture is when a monkey sits slumped with their head at or below the shoulders [19]. It is seen in 7% of indoor-housed rhesus macaques, although estimates are higher if only single-housed animals are considered (e.g., 19%; [20]). From a welfare perspective, this posture is concerning since it may reflect a depressive-like state, sharing a similar etiology with human depression [21,22], including chronic stress (e.g., peer separations: [23]; social stress: [24,25]; long-term social isolation: [20,26]) and/or early life adversity (e.g., maternal separation: [27,28,29]). Other inactive behaviours, such as leg-lifting and hanging, have not been researched to the same extent as other ‘postural’ abnormal behaviours.

Despite the superficial morphological similarities between the behaviours grouped within these four subcategories, researchers have not systematically studied whether they co-occur with each other more often than expected by chance, also known as comorbidity [30]. As such, if behaviours within the same subcategory are comorbid, then the subcategory shows construct validity—i.e., the subcategory accurately measures the behaviours it claims to assess. One form of construct validity is convergent validity, which is met when constructs hypothesized to be related are indeed found to be similar to one another, thus predicting positive relationships between different behaviours in the same subcategory.

One way to assess comorbidity is to run a principal component analysis (PCA). This statistical method identifies common factors among different variables to then create a smaller number of groups (‘components’) that share a similar variance [31]. Each variable either has a positive or negative relationship to the overall component, such that a component can be made up of variables that positively or negatively covary (e.g., [32]). In laboratory monkeys, PCAs have found abnormal behaviours to also load onto different components—but not always in ways predicted by the four hypothesized subcategories. For example, across 28 capuchin (*Sapajus* spp.) behaviours, at least four being ‘motor’ SBs (pacing, head-shaking, spinning, and bouncing), five components were found, which the authors concluded as representing different response styles to captivity [33]. One was composed of a high frequency of pacing (i.e., a positive loading) and a low frequency of self-grooming (i.e., a negative loading); another component involved a high frequency of head-shaking and spinning (i.e., both showed positive loadings), and a low frequency of ingesting urine, feces, or semen (i.e., a negative loading); and one involved high frequencies of bouncing and sexual display to humans (i.e., both showed positive loadings). Overall, this study demonstrated that the ‘motor’ subcategory is heterogeneous. Likewise, in infant rhesus macaques, a PCA revealed that floating limb and self-biting were positively loaded onto one component (despite traditionally being categorized as separate), while ‘motor’ SBs and ‘self-stimulation’ SBs were positively loaded onto another; rocking appeared to show negligible loadings with either component [34]. Finally, in adult rhesus macaques, another PCA found floating limb, self-injurious behaviours (including self-biting), and ‘motor’ SBs to all positively load onto the same component, while ‘self-stimulation’ SBs did not [13]. This suggests that some subcategories (at least ‘postural’ and ‘self-abuse’) may be too narrow and could be expanded. Most importantly, such results demonstrate how age affects the relationship between different abnormal behaviours and should thus be statistically controlled for in understanding these behaviours.

Since researchers have demonstrated that ‘abnormal behaviour’ is at best, an umbrella term [35], different forms should be analyzed separately when possible. However, this may not be practical as stated earlier, which highlights the value of creating subcategories. Nonetheless, the current subcategories have not been validated, and some PCA results suggest they may even be invalid (e.g., rocking not loading with other ‘motor’ SBs: [34]). This is problematic because there is a vast literature across different species showing how abnormal behaviours (particularly SBs) are heterogeneous. For example, abnormal behaviours are differentially affected by species-specific biology [36,37], drug and brain alterations [38,39,40,41], and environmental stimuli [42,43,44,45,46], while also demonstrating diverse welfare implications and risk factors [15,47,48,49,50,51,52]. Focussing on risk factors, Lutz et al. [48] found ‘motor’ SBs in laboratory baboons (*Papio hamadryas* spp.) to be predicted by a younger age when first individually housed, while ‘self-directed’ SBs were positively predicted by the number of blood draws and the time spent single-housed. Moreover, Lutz et al. [15] found that the duration of single housing only positively predicted ‘self-directed’ SBs such as eye-poking, hair-pulling, and self-biting in rhesus monkeys, while a negative relationship was found for ‘motor’ SBs such as body-flipping and swinging. Likewise, rearing type only predicted digit-sucking with nursery rearing increasing its risk compared to mother rearing, while not significantly predicting other SB forms. They also found the number of blood draws to positively predict eye-poking and self-injurious behaviour, while it negatively predicted pacing. These findings demonstrate heterogeneity between and within subcategories since several behaviours had different risk factors.

Given that abnormal behaviours are important cage-side welfare indicators, pooling heterogeneous ones together may reduce their precision in tackling husbandry refinements. For example, in fur-farmed mink (*Neovison vison*), Polanco et al. [45] found that neighbour proximity only triggered stereotypic scratching on cage walls, and this SB was less likely to be reduced by physical enrichments than other SBs. Yet, despite this, we currently lack clear guidelines on which behaviours can be appropriately pooled in laboratory monkeys. We resolve this problem by investigating whether four previously used subcategories (‘motor’, ‘self-stimulation’, ‘postural’, and ‘self-injurious’), totalling 19 abnormal behaviours, have convergent validity (i.e., behaviours in the same subcategory should covary with each other). Our work is also the first study to look at the relationship between 19 different abnormal behaviours in laboratory monkeys—to date, the most that have ever been investigated. Moreover, we aimed to provide researchers with alternative methods to a PCA that they can use to validate their own behavioural categories, if either replicating this work or working with a different species. In this study, we used logistic regression models, testing the prediction that the most prevalent behaviour of a subcategory co-occurs with other behaviours in the same subcategory. Where results did not support this prediction, we then sought to create new, valid subcategories that could be used instead. These analyses also controlled for sex and age to parallel human research on comorbidity (e.g., [53,54]) and to ensure our findings are generalizable to other laboratory monkey populations within the age range of our sample (i.e., young adults).

## 2. Materials and Methods

### 2.1. Ethical Approval

Data were obtained from the California National Primate Research Center’s (CNPRC) electronic database. As such, no living animals were directly used for this study. Nonetheless, all subjects were cared for in compliance with protocols approved by the Institutional Animal Care and Use Committee at the University of California Davis and followed the requirements of the Animal Welfare Act of the US Department of Agriculture [56].

### 2.2. Subjects and Housing

The study population comprised 1327 indoor-housed rhesus macaques from the CNPRC (54% female and mean age of 7.72 years, SD = 5.41), animals for which there were data on individual forms of abnormal behaviour. When observed, subjects were typically single-housed (*n* = 685) in standard cages (0.58 m × 0.66 m × 0.81 m) or continuously paired with another monkey in an adjacent cage (*n* = 314). Alternatively, they were intermittently paired with another monkey in an adjacent cage during the day but separated overnight (*n* = 405). Another type of housing used was grate-pairing (continuously or intermittently: *n* = 106) which involved a mesh grate between cages that allowed tactile, but not full contact. Note that the number of subjects in different housing adds to more than 1327 because some individuals were caged in more than one type of housing during the study period. All cages were on a 12-h light–dark cycle and contained a mixture of foraging and/or occupational enrichment (e.g., plastic toys).

### 2.3. Behavioural Observations

Data on 19 abnormal behaviours (Table 1) were collected for ten months using ‘abnormal behaviour scans’ (ABS) and ad hoc opportunistic observations. Opportunistic observations involved behavioural observations that occurred outside formal ABS (typically for rare behaviours such as ‘self-abuse’, Table 1). ABS involved trained primate technicians (inter-rater observer reliabilities of >85%) who observed 4–16 indoor-housed animals at a time for 5 min using 1–0 sampling with 1-min intervals, such that an animal would obtain a score between 0 and 5 for each behaviour (see ethogram). Both types of observations occurred several times throughout the year, but not very often (with the range of ABS and opportunistic observations over the year across animals being 1–14; median = 2; interquartile range = 1–4).

### 2.4. Statistical Analyses

Over 1000 subjects (*n* = 1183) showed at least one abnormal behaviour. We incorporated an estimate of data quality into the models to control for any potential bias (especially towards Type II errors) introduced by sparse sampling. Specifically, three-quarters of animals were observed only four times or less, and the number of different abnormal behaviours recorded increased with the number of observations made (Spearman *rho* = 0.60, *p* < 0.001). As such, we classified the frequency of scans into three levels: ‘rarely’ observed subjects were scanned only once (*n* = 412), ‘moderately’ observed subjects were scanned two or three times (*n* = 395), and ‘frequently’ observed subjects were scanned four or more times (*n* = 376). Data were analyzed with STATA 14.2 (StataCorp, College Station, TX, USA).

Before hypothesis testing, we explored how age, sex, and scanning level affected the odds of seeing each type of abnormal behaviour using univariable logistic regression models. For these analyses, all tests were two-sided with a significance level of 5% (i.e., alpha = 0.05).

Next, to investigate the convergent validity of the four subcategories, we first attempted a PCA, but sampling adequacy and linear relationships were not met after converting count data to frequencies (total counts of behaviour/total number of scans) [57]. As such, each abnormal behaviour was converted into presence/absence (1/0) across all scans. ‘Presence’ means the animal showed the behaviour at least once during the entire observational period, and ‘absence’ means the animal never showed the behaviour (at least during observations). Having dichotomous outcomes, we chose to use logistic regressions over a multiple correspondence analysis because the output of the latter tends to be subjective (e.g., involving interpretation of a two-dimensional coordinate plot) and cannot control for confounding variables. Here, the most prevalent behaviour was the dependent variable, and each secondary behaviour was explored separately as an independent variable. As is common in comorbidity research (e.g., [53,54]), all main models controlled for age (continuous) and sex (categorical), in addition to scanning level (categorical). However, we also present unadjusted results for comparison. We also explored the interaction between the secondary behaviour and scanning level. The linearity assumption for age was assessed graphically by generating locally weighted regression curves (lowess) with the outcome on the log odds scale and by assessing the significance of the addition of a quadratic term to the model.

Convergent validity was met if there was a significant and positive main effect of all other behaviours in the same subcategory. If the interaction between the secondary behaviour and scanning level was significant, then contrast tables were used to compare different covariate patterns of the interacting variables. For significant interactions, convergent validity was met if the positive relationship was present in frequently observed animals (not just detectable in rarely and/or moderately scanned animals, due to concerns about poorer data quality here). Since we were testing the hypothesis that behaviours in the same subcategory co-occur, we expected a positive association. Due to the directional nature of the hypothesis, one-tailed *p*-values were reported for these associations with a significance level of 5% [58].

Since none of the subcategories met the requirements of convergent validity (see Results below), we then investigated which individual behaviours were positively associated with each other, to create new valid subcategories. Consistent with the steps above, if there was a significant and positive main effect of the predictor behaviour on the outcome behaviour, then they were pooled together as a new subcategory. For interactions with scanning frequency, behaviours were only pooled together if the significant main effect was found in the frequently observed group. For any behaviours remaining in the original subcategory, we repeated the process using the next most prevalent behaviour as the dependent variable and the remaining behaviours as the independent variables, while including their interaction with scanning frequency, and controlling for age, sex, and scanning level (as above). Again, any behaviours that were significant and positively associated with each other were pooled into new subcategories, while any remaining behaviours were kept as individual behaviours. To parallel the analyses that tested the validity of the four subcategories, we again reported one-tailed *p*-values for these associations.

We further investigated whether these subcategories and behaviours were positively related to each other using similar models as above. These results are reported in a matrix table, showing the odds ratio for when each behaviour or subcategory was the dependent variable. Here, we again then pooled behaviours that showed a significant and positive association with each other. If there was a case in which A significantly and positively predicted behaviours B and C, but the positive relationship between B and C was non-significant, then these were still pooled for practical usefulness. However, if the relationship between B and C was negative, then behaviour C was not pooled with behaviours A and B.

Model fit was assessed with Hosmer–Lemeshow goodness-of-fit tests (*p* < 0.05 indicating a model does not fit the data). If model fit was not met, then we used robust standard errors to estimate *p*-values and 95% CIs. However, if this made the *p*-value smaller, the larger, more conservative *p*-value was reported. For all multivariable models, possible multicollinearity was assessed using variance inflation factor (VIF). A VIF value > 10 suggests the presence of multicollinearity, but this was not detected (the exception being the quadratic term of age, when included).

## 3. Results

### 3.1. Descriptive Statistics

**Figure 1:**
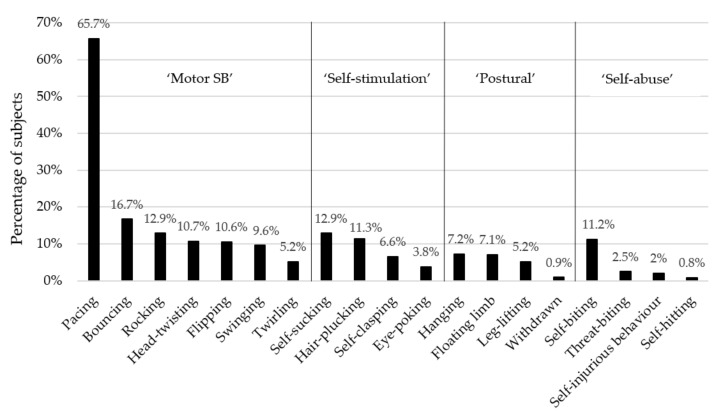
Prevalence of subjects displaying the 19 abnormal behaviours (*n* = 1183 rhesus macaques).

On average, each individual monkey performed two different types of abnormal behaviour (range 1–9). For ‘motor’ SB, pacing was the most common form. For ‘self-stimulation’, self-sucking was the most prevalent form. For the ‘postural’ subcategory, hanging was the most prevalent behaviour. For ‘self-abusive’ behaviours, self-biting was the most common form; see Figure 1 for details.

Regarding the effects of demographic variables and scanning level, results are as follows. Age was positively and significantly associated with rocking, hair-plucking, and being withdrawn. In contrast, age was negatively and significantly associated with pacing, bouncing, head-twisting, flipping, swinging, twirling, self-sucking, hanging, and leg-lifting. Males also showed significantly higher odds of bouncing, self-sucking, self-biting, and threat-biting compared to females. Moreover, receiving more behavioural scans often significantly increased the odds of abnormal behaviours being reported (although not always; see Table 2).

### 3.2. Do the Hypothesized Subcategories of Abnormal Behaviours have Convergent Validity?

Starting with the ‘motor’ SB subcategory, scanning level was part of a significant interaction for only two behaviours: flipping and rocking. However, these interactions revealed no significant positive relationships with pacing. Moreover, based on the unadjusted analyses, twirling and swinging both had a significant positive association with pacing. However, these relationships were no longer significant after controlling for age, sex, and scanning level. Lastly, we found that head-twisting significantly increased the odds of pacing, even after controlling for potential confounders; see Table 3.

Regarding the ‘self-stimulation’ subcategory, scanning frequency did not significantly interact with any of the predictor variables. Furthermore, the positive relationship between self-sucking and eye-poking was not statistically significant, while the positive relationship between self-sucking and self-clasping was no longer significant after controlling for sex, age, and scanning frequency. Lastly, self-sucking did not positively covary with hair-plucking; see Table 4.

For the ‘postural’ subcategory, scanning frequency did not significantly interact with any of the predictor variables. Hanging was not positively associated with floating limb, leg-lifting, nor withdrawn (Table 5).

Lastly, for the ‘self-abuse’ subcategory, scanning frequency only significantly interacted with threat-biting. Here, the odds of self-biting were significantly higher in rarely scanned animals who also showed threat-biting, but this was not significant in the other scanned groups. This result is likely a Type I error as these data are the least reliable. Furthermore, the odds of self-biting were significantly higher if monkeys showed self-hitting and self-injurious behaviours; see Table 6.

### 3.3. Forming New Subcategories

Based on the first set of models, investigating the ‘motor’ subcategory (Table 3), head-twisting was pooled into a new subcategory with pacing, given their significant positive association. Bouncing was the most prevalent behaviour remaining in the ‘motor’ subcategory. Following the same methods as above, we investigated whether the remaining behaviours predicted bouncing. Both rocking and swinging significantly increased the odds of bouncing (ORs = 2.60, *p* < 0.05, see Table A1). Thus, they were pooled to form a new subcategory. Furthermore, twirling and bouncing positively covaried, as did flipping and bouncing, but these relationships became negative and/or non-significant after controlling for sex, age, and scanning level (Table A1). Thus, flipping and twirling remained in their own subcategories since they did not significantly positively covary with pacing (Table 3), nor with bouncing (Table A1).

Furthermore, in the second set of models, investigating the ‘self-stimulation’ subcategory (Table 4), self-sucking did not show a significant positive association with any other behaviour after controlling for potential confounders. Since hair-plucking was the most prevalent behaviour remaining in the former ‘self-stimulation’ subcategory, we investigated whether self-clasping and eye-poking were positively associated with it. Given the absence of significant positive relationships (Table A2), all four ‘self-stimulation’ behaviours were split into individual subcategories.

The third set of models, investigating the ‘postural’ subcategory (Table 5), revealed no positive associations with hanging. Floating limb was the next most prevalent behaviour in this subcategory. Results showed it to be positively associated with leg-lifting (OR = 8.45, *p* = 0.03), but not with withdrawn (see Table A3). As such, floating limb and leg-lifting were pooled, while hanging and withdrawn remained as individual behaviours.

The last set of models, investigating the ‘self-abuse’ subcategory (Table 6), revealed that self-biting was significantly positively associated with self-hitting and self-injurious behaviours. Thus, these three behaviours were pooled. Threat-biting remained on its own since it did not consistently show a significant positive relationship with self-biting among frequently scanned animals.

### 3.4. Are Any of the Newly Created Subcategories Related to Each Other?

We further assessed whether the newly identified subcategories and behaviours could be pooled. The subcategory ‘self-bite/self-hit/self-injurious behaviour’ was significantly positively associated with self-clasp and with ‘floating-limb/leg-lift’, even after controlling for sex, age, and scanning level. ‘Floating limb/leg-lift’ and self-clasp also appeared positively associated with each other, but this relationship ceased being statistically significant after controlling for potential confounders (Table 7 and Table A4). Given the robust positive associations between self-clasp, ‘floating-limb/leg-lift’, and ‘self-bite/self-hit/self-injurious behaviour’, these were then pooled to form a new subcategory (see final subcategory formation in Table 8).

Furthermore, based on adjusted associations, there was a significant and positive relationship between twirling and the three following behaviours and subcategories: ‘bounce/rock/swing’, ‘pace/head-twist’, and hanging. Moreover, ‘bounce/rock/swing’ was significantly and positively associated with hanging (again even after adjusting for potential confounders). However, ‘pace/head-twist’ showed negative relationships with both ‘bounce/rock/swing’ and hanging (Table 7 and Table A4). Thus, only twirling, ‘bounce/rock/swing’, and hanging were pooled together, while ‘pace/head-twist’ remained as is (Table 8).

Additionally, adjusted associations found a significant and positive relationship between eye-poking and flipping; thus, they were pooled to form a new subcategory. However, other positive relationships with flipping (i.e., ‘floating-limb/leg-lift’, twirling, and hanging) and eye-poking (i.e., ‘floating-limb/leg-lift’, ‘self-bite/self-hit/self-injurious behaviour’, and threat-biting) were no longer significant after controlling for potential confounders, and were thus not considered robust.

On the other hand, some behaviours did not show positive and significant relationships with any other behaviour, and were thus categorized individually. These included self-sucking, hair-plucking, and withdrawn (Table 7). For example, self-sucking appeared positively associated with ‘self-bite/self-hit/self-injurious behaviour’, ‘floating-limb/leg-lift’, and self-clasping, but these relationships were no longer significant after controlling for potential confounders (see Table 7 and Table A4). Again, these relationships were thus not considered robust. Moreover, threat-biting remained as an individual behaviour because the significant positive relationship between threat-biting and ‘floating limb/leg-lift’ was only found in rarely scanned animals (Table 7 and Table A4). Additionally, while ‘self-bite/self-hit/self-injurious behaviour’ was positively associated with threat-biting (Table A4), this relationship was not significant when ‘self-bite/self-hit/self-injurious behaviour’ was the dependent variable (Table 7). For the final subcategories created, see Table 8.

## 4. Discussion

The hypothesis that four existing abnormal behaviour subcategories have convergent validity was not supported. This indicates that researchers have been erroneously combining behaviours that do not statistically co-occur, while also keeping some behaviours separate when they should be pooled. Specifically, the ‘motor’ subcategory proved to be too broad, as ‘motor’ SBs were eventually split across three different subcategories during final validation (Table 8). That the ‘motor’ SB group was too heterogeneous was also previously suggested in different studies applying a PCA (e.g., [34]) and assessing risk factors (e.g., [15]; see also Table 1). In contrast, the original ‘postural’, ‘self-stimulation’, and ‘self-abuse’ subcategories were all found to be too narrow: each excluded other behaviours that proved to be comorbid. In prioritizing physical similarity (e.g., ‘postural’) and putative function (e.g., ‘self-stimulation’), researchers had thus previously used descriptors that are intuitive to use, but that failed to capture or genuinely pool comorbid behaviours. Alternatively, past work indicating comorbidity between different behaviours may have been finding spurious correlations—behaviours just *appearing* to co-occur simply because they were jointly affected by age and/or sex. Our current work presents comorbid behaviours *after* adjusting for such confounders, as is common in human comorbidity research (e.g., [53,54]). This strategy thus yielded subcategories that should have greater generality, being valid across ages and sexes.

Indeed, using our large sample size and recognizing the value of pooling behaviours for practical welfare assessments, we created four new subcategories. These subcategories contain behaviours that superficially appear heterogeneous: they vary in their degrees of rhythmicity (from inactive postures to repetitive movements: e.g., see new ‘bouncing, rocking, swinging, twirling and hanging’ subcategory in Table 8), in the body parts involved (from involving one limb to the entire body: e.g., see new ‘flipping and eye-poking’ subcategory in Table 8), and in the amount of cage space used (from pacing along cage walls to head-twisting in one spot: see new ‘pacing and head-twisting’ subcategory in Table 8). However, despite this, the new subcategories have genuine convergent validity.

Four behaviours were also now placed into their own solo subcategories (self-sucking, hair-plucking, threat-biting and being withdrawn: Table 8); these appeared to be unique, as if sharing no underlying causal factors with other abnormal behaviours. The etiology of these four behaviours, and our new subcategories, is now discussed in more detail.

### 4.1. Self-Sucking

Self-sucking showed positive associations with other ‘self-stimulation’ behaviours (e.g., self-clasping and eye-poking) and ‘self-abuse’ behaviours (e.g., self-bite, self-hit, and self-injurious behaviour), but these relationships were no longer statistically significant after controlling for age, sex, and scanning level. Since such associations were likely confounded with these variables, we thus recommend categorizing self-sucking separately.

Regarding its etiology, self-sucking could be a redirected behaviour that would have been performed on the dam during infancy. This is supported by Lutz et al. [15], who found that nursery rearing (vs. mother rearing) increased the risk of digit sucking, but not other abnormal behaviours, and Cross and Harlow [59], who found that toe sucking drastically decreased with age. Self-sucking could also be a response to agonistic events: one study found that threatening stimuli (e.g., presence of a catching glove) triggered digit-sucking in nursery reared rhesus macaques [59]. Thus, self-sucking appears to be specific to nursery reared infants and juveniles. Our analyses further show that self-sucking is more common among males and less common with age.

### 4.2. Hair-Plucking

Hair-plucking increased with age, while it was not significantly predicted by sex in our sample. Further, it was not found to be comorbid with any other activity: it negatively correlated with almost all other subcategories and individual behaviours. Such negative relationships could signify distinct mechanisms. Indeed, hair-plucking has been hypothesized to be caused by frustrated motivations to groom conspecifics [36]—a potential explanation unique to this behaviour. Hair-plucking may also reflect a primate ‘coping’ mechanism. Prior work has found that hair-plucking, pooled with nail-biting and digit-sucking (a combination we were unable to validate with this dataset) is negatively correlated with fecal corticoids following an acute stressor [47]. Likewise, hair-pulling in humans is self-reported to be stress-reducing [60]. The coping hypothesis can now be tested in future research (see below), and more effectively so, if hair-plucking is not pooled with other unrelated self-directed behaviours.

### 4.3. Threat-Biting

Threat-biting was more prevalent in males, while it was not significantly associated with age. We found that the odds of ‘self-abuse’ behaviours, floating limb, leg-lift, and eye-poking were 2–9 times higher if monkeys also showed threat-biting. However, these positive associations were either only seen in rarely scanned animals or they were not always statistically significant (e.g., after adjusting for potential confounders). This warrants further research exploring whether these behaviours truly co-occur. For now, we recommend categorizing this behaviour on its own, which is how the CNPRC currently categorizes it. Indeed, they view threat-biting as distinct from other self-abusive and self-directed behaviours, regarding it as a signal of aggression since it appears to be directed at other animals [61].

### 4.4. Being ‘Withdrawn’

‘Withdrawn’ behaviour positively covaried with age, while it is not significantly different between males and females in our sample. Further, it did not show any significant positive association with other behaviours. This maps onto other findings that suggest ‘withdrawn’ has distinct mechanisms than other behaviours. For one, it is the only abnormal primate behaviour that seems to increase with pain. Monkeys who were euthanized due to being severely ill spent significantly less time in normal stationary positions and more time displaying clinical signs, including a hunched posture, compared to monkeys who were ill yet survived [62]. As mentioned earlier, withdrawn behaviour has also been suggested to indicate a depressive-like state [19], which is corroborated by human evidence: hunched postures are more likely to be seen in clinically depressed individuals than controls [63,64,65]. Thus, future work should test the hypothesis that a hunched posture reflects a depressive-like state, while other abnormal behaviours do not.

### 4.5. Self-Biting and Correlates

The first of our four new subcategories combines most ‘self-abuse’ behaviours with floating limb, leg-lifting, and self-clasping, thereby mixing elements from three of the four original subcategories. Prior work supports this new subcategory as floating limb predicts the later development of self-biting in laboratory monkeys [18,34]. Moreover, floating limb and self-clasping time budgets are positively correlated in another species and facility type: zoo-housed stump-tailed macaques (*Macaca arctoides*) [66].

Furthermore, the comorbidity of these behaviours could reflect shared social deprivations or vulnerabilities to such management. Thus, self-clasping (but also self-sucking) is more common in infants raised in total isolation [67], thereby suggesting that some aspects of rearing involving maternal and/or peer separation can trigger it. Likewise, floating limb and leg-lifting are increased by nursery rearing (reviewed by [68])—a risk factor reported for other types of abnormal behaviours too (e.g., self-biting and ‘motor’ SBs [5]). Moreover, the behaviours pooled here may also have a shared ability to help animals cope with sub-optimal treatment. For instance, self-biting reduces heart rate to baseline levels ([7]; reviewed more in [69]). Nonetheless, which of these causal factors best explains this subcategory requires more research.

### 4.6. Bouncing and Correlates

Our second newly created subcategory is made up of bouncing, rocking, swinging, twirling, and hanging. In a prior PCA, rocking formed its own component when removed from the ‘motor’ SB subcategory [34], again highlighting the lack of validity of this subcategory. However, other motor behaviours were not individually assessed in that PCA, so it is possible that rocking could have loaded onto the same component as bouncing, twirling, and swinging.

Regarding underlying causes, maternal deprivation studies have again found postpartum maternal absence to trigger the development of rocking in laboratory infant monkeys [70]—a pattern also seen in institutionalized human children [71]. As well, experiments using stationary or moving artificial surrogate mothers found that only monkeys raised with the stationary surrogate developed rocking in infancy, while both groups developed self-clasping (or self-sucking). Hence, this suggests that a lack of movement and proprioceptive stimulation may be key for rocking [72]. Similarly, researchers have hypothesized that bouncing and rocking, seen in humans from both control and clinical populations [73,74], are likely caused by poor sensorimotor integration, which in turn, may be produced by immature motor brain regions during early development or from motor dysfunction commonly seen in developmental disorders [75]. Likewise, twirling in humans is typically only seen in those with developmental disorders (e.g., autism), therefore most likely stemming from motor dysfunction too [76].

Hanging and swinging remain a puzzle as they would not appear to align with these ideas. Since both hanging and swinging involve holding onto the ceiling cage bars, it is possible that monkeys hang from the cage bars after a bout of swinging (or vice versa). However, this needs to be assessed with future research describing the developmental sequence of abnormal behaviours in laboratory monkeys. It could also be that hanging and swinging manifest from a frustrated motivation to tree swing (see future work below).

### 4.7. Pacing and Head-Twisting Subcategory

Our third new subcategory is comprised of pacing and head-twisting, which were part of the original broad ‘motor’ SB subcategory. While ‘pace/head-twist’ also positively covaried with twirling, it negatively covaried with hanging and ‘bounce/rock/swing’. Thus, researchers may pool pacing, head-twisting, and twirling together if they do not observe hanging, bouncing, rocking, and swinging. However, if all the behaviours are observed, then we recommend following the scheme in Table 8.

Regarding etiology, pacing time-budgets across diverse species of zoo primates are positively associated with daily distances travelled in the wild, suggesting that pacing may stem from a thwarted motivation to range [35,36]. This mechanism could also explain head-twisting which may resemble scanning motions required for successful arboreal travel in the wild. Interestingly, pacing and head-twisting have also been reported as co-occurring in Carnivora too [77,78]. Furthermore, other hypotheses regarding the causal factors of pacing include boredom and basal ganglia dysfunction (see review by [35]).

### 4.8. Flipping and Eye-Poking Subcategory

Our final new subcategory is formed of flipping and eye-poking, which originally were pooled with ‘motor’ or ‘self-stimulation’ behaviours, respectively. Despite this, one study found distinct risk factors for them: years single-housed increased the risk for eye-poking, while it decreased the risk for flipping [15]. This suggests that single housing is not a common trigger for both behaviours. Instead, eye-poking may serve a visual stimulation function, similar to intellectually disabled children who eye-poke [79]. Additionally, research on laboratory deer mice (*Peromyscus maniculatus*) suggests that flipping may develop from other SBs [80,81]. Whether these two behaviours develop together and/or serve a type of sensory stimulation in laboratory monkeys requires more research.

### 4.9. Strengths and Limitations

Strengths of this study include our broader selection of behaviours compared to past macaque studies, our large sample size, and our use of logistic regression models. Such models allowed us to model binary outcomes, which did not require data to be continuous, and permitted analysis of rare behaviours. A limitation of PCA is that it is not appropriate for right-skewed data (e.g., containing many zeros due to rarity), since it relies on linear relationships. As such, prior PCAs have omitted rare behaviours (i.e., those occurring <1% of the time: [32]). Nonetheless, while we acknowledge that using binary instead of continuous data may be less precise, any data loss experienced with our dichotomization was negligible in this particular dataset since the median number of scans was only two. Furthermore, a PCA can combine components with both positive and negative loadings, whereas our approach specifically allowed us to identify positive relationships and thus comorbidity. Finally, our logistic regression models controlled for age and sex, while prior PCA work on laboratory rhesus macaques pooled males and females [13,34]. This is important as age and sex affected the likelihood to display half or more of these behaviours, as previously documented [5,6,7,9,15,16,20,82]. Consequently, as mentioned earlier, some previous components identified could have merely reflected the confounding effects of sex and age. Consistent with this, we found that some relationships between different behaviours were no longer significant after adjusting for these confounders.

Some limitations include how the lack of significant relationships could reflect Type II errors, especially given the limited data quality. For example, since the data were limited to catching co-occurrences during a 5-min observation window, it is possible that other co-occurrences did happen, but at other times of the day. Likewise, some behaviours may co-occur when human observers are not present—a limitation applicable to all studies reliant on live data collection. This is particularly relevant for withdrawn postures, which are more likely to be seen via video observations than live human observations [20]. Moreover, some results were only found in moderately and frequently scanned animals, suggesting that more frequent scans are likely to capture the true relationship between different abnormal behaviours than infrequent scans. As well, this study aggregated scans of each subject into a single binomial score, thereby omitting within-subject variation. As such, the final subcategories are not suitable to test hypotheses about intra-individual effects. Moreover, it is possible that our multiple testing created some Type I errors. This indicates that our findings should be replicated in other populations to ensure our recommended subcategories are truly applicable to other laboratory rhesus macaques. Lastly, although our findings are generalizable to other young adult monkey populations, the final subcategories cannot be generalized to very young or old laboratory monkeys (i.e., populations that were outside our age range).

### 4.10. Future Research

We suggest several research avenues from this new scheme, including ones pertaining to risk factors, correlates, and treatments. However, all of these assume that our results can be replicated in other populations, an important next step. In addition, researchers who replicate this study should also further refine their ethogram to ensure they are not mistakenly capturing agitated locomotion (i.e., ‘moving fast between locations with a stiff un-relaxed gait’ [83]) with pacing.

Building on this, several new research questions then arise. First, these distinct subcategories and behaviours are hypothesized to have different risk factors. Based on the work reviewed above, we hypothesize that early life experiences involving maternal deprivation are risk factors for self-sucking, self-biting and its correlates, and bouncing and its correlates. Additionally, restricted ranging (perhaps inferred by indoor housing length) is especially likely to be a risk factor for the ‘pacing and head-twisting’ subcategory, whereas single housing is suggested to be a key risk factor for hair-plucking. Moreover, a history of chronic stress (perhaps inferred by number of research projects, location moves, and pair separations) would likely increase the risk of a withdrawn posture.

Second, these newly validated subcategories and behaviours are hypothesized to have distinct correlates or consequences. For instance, the ‘coping’ hypothesis can be tested by assessing the time spent performing different subcategories before, during, and after an aversive event (e.g., [83]), and seeing if behavioural changes covary with changes in heart rate and cortisol. Based on the literature above, it is hypothesized that self-biting and its correlates would help animals cope with stress. Moreover, the motor dysfunction hypothesis can be tested by studying the associations between the different validated subcategories and “perseveration”—a neuropsychological measure of forebrain function that measures the amount of inappropriate repetition of responses in a task [84]. In monkeys, this can be tested with an operant task where subjects are rewarded for choosing one of two holes in an apparatus mounted in front of their home cage (e.g., [47]). Here, it is hypothesized that only bouncing and its correlates would covary with perseveration. Researchers can also test the hypothesis that a withdrawn posture reflects a depression-like state by seeing how it covaries with other diagnosable symptoms of clinical depression in monkeys [85]. As well, future research can investigate if threat-biting is a form of aggression by seeing how it covaries with agonistic behaviours such as cage-shaking or fear grimaces.

Third, these distinct subcategories and behaviours could also benefit from specifically tailored changes to housing and husbandry. For example, ‘pacing and head-twisting’ may best be managed with access to outdoor housing, foraging enrichments, and/or increased space. Similarly, if ‘flipping and eye-poking’ derive from a lack of sensory stimulation, then sensory enrichment, or digital enrichment (e.g., involving the use of touchscreens) may decrease them. Moreover, hair-plucking, self-sucking, threat-biting, and self-biting (along with its correlates) may benefit from social housing and/or anxiolytics. Furthermore, a withdrawn posture may be treated with antidepressants. In contrast, if bouncing and its correlates reflect underlying neurological changes, then this subcategory may be the hardest to treat.

In conclusion, we have demonstrated an effective approach to assess convergent validity that can be adopted by researchers working with other species, in addition to validating an abnormal behaviour ethogram in laboratory monkeys. Based on our new validated subcategories, we propose several key hypotheses for further study regarding the treatment and management of abnormal behaviours.

## Figures and Tables

**Table 1:** Ethogram and summary of prior work assessing comorbidity and convergent validity of abnormal behaviours in laboratory rhesus macaques.

Behavioural Subcategory	Individual Behaviour	Description	Comorbid with Other Behaviours? *	Does Prior Work Support or Contradict the Subcategory’s Construct Validity?
‘Motor’ stereotypic behaviours [5,9,13]	Pace	Walking back and forth or in a circular pattern, for at least three repetitions or 5 or more seconds.	‘Self-stimulation’ SBs [34]; cf. [13]; floating limb [13]; cf. [34]; self-injurious behaviours [13]; cf. [34], including self-biting [5].	Individual ‘motor’ behaviours showed distinct risk factors [15], and rocking formed its own component in a PCA [34], thereby suggesting this subcategory does not have construct validity.
	Flip	Turning a somersault, usually in a backwards fashion, for at least three repetitions or 5 or more seconds.		
	Twirl	Swinging in a circle or spinning, for at least three repetitions or 5 or more seconds.		
	Swing	Moving back and forth suspended from above, for at least three repetitions or 5 or more seconds.		
	Bounce	Moving jerkily, usually up and down, for at least three repetitions or 5 or more seconds.		
	Head-twist	Moving or lifting the head with a sudden motion, for at least two repetitions or 5 or more seconds.		
	Rock	Moving back and forth or from side to side, especially gently or rhythmically, for at least three repetitions or 5 or more seconds.		
‘Self-stimulation’ [13,15,34]	Self-clasp	Embracing or hugging oneself, for at least 15 s.	‘Motor’ SBs [34]; cf. [13].	Individual ‘self-stimulation’ behaviours showed distinct risk factors [15]; thereby suggesting this subcategory does not have construct validity.
	Hair-pluck	Excessive pulling of one’s hair; often leads to over-groomed appearance.		
	Self-suck	Sucking various parts of one’s body including digits, tail, and male genitalia.		
	Eye-poke	‘Saluting’ gesture of hand over eye; often involves a digit (frequently the thumb) being pressed into the eye-socket.		
‘Postural’ [13]	Hang	Hanging from the cage ceiling with 1–4 limbs, for at least 15 s.	No prior data.	No prior data comparing individual forms.
	Floating limb	Arm or leg being lifted seemingly without the animal’s knowledge; sometimes used to self-groom as though the animal is being groomed by another; often results in self-biting when animal sees limb.	Self-biting [13,34]; ‘motor’ SBs [13]; cf. [34].	
	Leg-lift	Arm or leg reaches around the back of the body or is propped on the back, for at least ten seconds.	No prior data.	
	Withdrawn	Facing a corner for an extended period of time; usually acting socially withdrawn, for at least 30 s.	No prior data.	
‘Self-Abuse’ [13,55]	Self-bite	Biting oneself; usually involves biting arms, legs, shoulders or genitals.	Floating limb [13,34]; ‘motor’ SBs [5,13]; cf. [34].	Self-biting and self-injurious behaviour share the same risk factors [15], therefore supporting this subcategory’s validity (although other forms were not assessed).
	Threat-bite	Biting hand, wrist, or forearm while staring at an observer, mirror, or conspecific in a threatening manner.		
	Self-hit	Striking oneself forcefully.		
	Self-injurious behaviour	Self-biting, scratching, or some other form of self-mutilation which results in injury. If the self-biting incident did not result in injury, then it was categorized as ‘self-biting’.		

* cf. is used to denote studies in which the behaviours in question are not found to be comorbid with each other, unlike the other studies cited.

**Table 2:** Univariable models for the relationships between each abnormal behaviour and three potential predictors: age, sex, and scanning level ^1^.

Independent Variable/Dependent Variable	Age			Male Sex (Referent = Female)			Moderate Scanning Level (Referent = Rare)			Frequent Scanning Level (Referent = Rare)		
	Odds Ratio	95% CI	*p*-Value	Odds Ratio	95% CI	*p*-Value	Odds Ratio	95% CI	*p*-Value	Odds Ratio	95% CI	*p*-Value
Pacing	0.97	0.95–0.99	**0.004**	0.80	0.63–1.02	0.076	3.00	2.25–4.00	**<0.001**	9.17	6.39–13.14	**<0.001**
Bouncing	0.95	0.92–0.97	**<0.001**	1.81	1.32–2.47	**<0.001**	1.72	1.16–2.55	**0.007**	1.92	1.30–2.84	**0.001**
Rocking	1.03	1–1.06	**0.031**	1.33	0.94–1.86	0.106	2.78	1.69–4.58	**<0.001**	3.70	2.27–6.02	**<0.001**
Head-twisting	0.93	0.89–0.96	**<0.001**	1.20	0.83–1.73	0.338	2.74	1.48–5.09	**0.001**	6.59	3.71–11.71	**<0.001**
Flipping	0.80	0.75–0.85	**<0.001**	1.04	0.72–1.50	0.844	1.72	1.01–2.93	**0.045**	3.25	1.99–5.33	**<0.001**
Swinging	0.84	0.80–0.89	**<0.001**	1.03	0.70–1.52	0.890	1.54	0.89–2.68	0.124	3.02	1.82–5.01	**<0.001**
Twirling	0.92	0.87–0.97	**0.004**	1.38	0.83–2.32	0.216	1.64	0.76–3.55	0.209	3.62	1.81–7.26	**<0.001**
Self-sucking	0.64	0.59–0.70	**<0.001**	1.83	1.29–2.58	**0.001**	0.97	0.63–1.51	0.904	1.53	1.02–2.31	**0.04**
Hair-plucking	1.17	1.13–1.20	**<0.001**	0.96	0.69–1.38	0.825	1.21	0.79–1.87	0.382	1.00	0.63–1.57	0.985
Self-clasping	0.99	0.95–1.03	0.77	0.86	0.54–1.36	0.513	1.10	0.59–2.03	0.765	1.91	1.09–3.35	**0.023**
Eye-poking	0.96	0.91–1.02	0.198	1.29	0.71–2.35	0.399	2.13	0.90–5.04	0.085	2.99	1.31–6.83	**0.009**
Hanging	0.93	0.88–0.97	**0.002**	1.35	0.87–2.10	0.184	1.13	0.65–1.97	0.659	1.33	0.78–2.29	0.297
Floating limb	1.02	0.98–1.05	0.393	1.46	0.94–2.28	0.096	2.14	0.95–4.82	0.067	8.00	3.90–16.41	**<0.001**
Leg-lifting	0.95	0.90–1.00	**0.036**	1.21	0.72–2.01	0.472	2.28	0.92–5.66	0.075	6.89	3.05–15.58	**<0.001**
Withdrawn	1.15	1.06–1.25	**0.001**	1.35	0.41–4.44	0.624	1.04	0.21–5.20	0.959	1.84	0.44–7.74	0.407
Self-biting	0.98	0.95–1.01	0.173	1.81	1.25–2.61	**0.002**	2.61	1.35–5.07	**0.005**	9.52	5.22–17.37	**<0.001**
Threat-biting	0.99	0.93–1.06	0.770	2.29	1.06–4.93	**0.035**	1.31	0.35–4.91	0.691	6.03	2.05–17.74	**0.001**
Self-injurious behaviour	0.99	0.92–1.06	0.793	1.58	0.70–3.59	0.272	0.69	0.19–2.47	0.571	2.61	1.00–6.88	0.051
Self-hitting	0.88	0.75–1.04	0.131	3.96	0.82–19.13	0.087	3.15	0.33–30.37	0.322	5.54	0.64–47.63	0.119

^1^ Two-tailed *p*-values are reported here. Significant *p*-values are bolded for emphasis.

**Table 3:** Logistic regression models for pacing presence (‘motor’ SB subcategory) ^1^.

Independent Variables	Unadjusted Odds Ratio and *p*-Value	Adjusted Odds Ratio *	95% CI *	*p*-Value *	Positively and Significantly Predicts Pacing? * *(Result the Same if Age, Sex and Scanning Level not Controlled for?)*
Flipping presence vs. absence (rarely scanned group)	0.33 (0.985)	0.31	0.11–0.87	0.987	No *(Unadjusted relationship is also negative)*
Flipping presence vs. absence (moderately scanned group)	1.28 (0.261)	1.07	0.49–2.34	0.436	No *(Unadjusted relationship is also positive and non-significant)*
Flipping presence vs. absence (frequently scanned group)	1.47 (0.201)	1.29	0.52–3.20	0.289	No *(Unadjusted relationship is also positive and non-significant)*
Twirling presence vs. absence	3.23 (<0.001)	1.66	0.48–5.70	0.210	No *(Unadjusted relationship is positive and significant)*
Swinging presence vs. absence	1.84 (0.005)	0.77	0.32–1.83	0.723	No *(Unadjusted relationship is positive and significant)*
Bouncing presence vs. absence	0.69 (0.989)	0.27	0.13–0.58	0.999	No *(Unadjusted relationship is also negative)*
Head-twisting presence vs. absence	4.77 (<0.001)	2.89	0.96–8.73	0.029	Yes *(Unadjusted relationship is also positive and significant)*
Rocking presence vs. absence (rarely scanned group)	0.11 (0.999)	0.12	0.03–0.49	0.998	No *(Unadjusted relationship is also negative)*
Rocking presence vs. absence (moderately scanned group)	0.41 (0.999)	0.45	0.25–0.79	0.997	No *(Unadjusted relationship is also negative)*
Rocking presence vs. absence (frequently scanned group)	0.64 (0.883)	0.66	0.32–1.36	0.871	No *(Unadjusted relationship is also negative)*

* Adjusted for sex, age, and scanning level. ^1^ One-tailed p-values are reported here. Significant *p*-values for adjusted positive relationships are bolded for emphasis.

**Table 4:** Logistic regression models for self-sucking presence (‘self-stimulation’ subcategory) ^1^.

Independent Variables	Unadjusted Odds Ratio and *p*-Value	Adjusted Odds Ratio *	95% CI *	*p*-Value *	Positively and Significantly Predicts Self-Sucking? * (Result the Same if Age, Sex and Scanning Level not Controlled for?)
Self-clasping presence vs. absence	2.52 (<0.001)	2.55	0.45–14.33	0.144	No *(Unadjusted relationship is positive and significant)*
Eye-poking presence vs. absence	1.48 (0.166)	2.24	0.08–64.81	0.319	No *(Unadjusted relationship is also positive and non-significant)*
Hair-plucking presence vs. absence ^2^	0.09 (0.999)	0.59	0.12–2.80	0747	No *(Unadjusted relationship is also negative)*

* Adjusted for sex, age, and scanning level. ^1^ One-tailed *p*-values are reported here. ^2^ Interaction not tested due to insufficient observations.

**Table 5:** Logistic regression models for hanging presence (‘postural’ subcategory) ^1^.

Independent Variables	Unadjusted Odds Ratio and *p*-Value	Adjusted Odds Ratio *	95% CI *	*p*-Value *	Positively and Significantly Predicts Hanging? * (Result the Same if Age, Sex and Scanning Level not Controlled for?)
Floating limb presence vs. absence ^2^	0.81 (0.675)	0.74	0.29–1.93	0.723	No *(Unadjusted relationship is also negative)*
Leg-lifting presence vs. absence ^2^	0.64 (0.764)	0.53	0.16–1.75	0.851	No *(Unadjusted relationship is also negative)*
Withdrawn presence vs. absence ^3^	0.84 (0.561)	0.84	0–5.19	0.561	No *(Unadjusted relationship is also negative)*

* Adjusted for sex, age, and scanning level. ^1^ One-tailed p-values are reported here. ^2^ Interaction not tested due to insufficient observations. ^3^ Based on a univariable analysis since there were too few observations to fit a multivariable model.

**Table 6:** Logistic regression models for self-biting presence (‘self-abuse’ subcategory) ^1^.

Independent Variables	Unadjusted Odds Ratio and *p*-Value	Adjusted Odds Ratio *	95% CI *	*p*-Value *	Positively and Significantly Predicts Self-Biting? * *(Result the Same if Age, Sex and Scanning Level not Controlled for?)*
Threat-biting presence vs. absence (rarely scanned group)	11.00 (0.022)	9.02	0.86–94.56	**0.034**	Yes *(Unadjusted relationship is also positive and significant)*
Threat-biting presence vs. absence (moderately scanned group)	3.00 (0.167)	2.76	0.29–25.84	0.188	No *(Unadjusted relationship is also positive and non-significant)*
Threat-biting presence vs. absence (frequently scanned group)	1.00 (0.495)	0.89	0.31–2.53	0.587	No *(Unadjusted relationship is also non-significant)*
Self-hitting presence vs. absence ^2^	6.48 (0.003)	4.04	0.96–16.98	**0.029**	Yes *(Unadjusted relationship is also positive and significant)*
Self-injurious behaviour presence vs. absence ^2^	7.19 (<0.001)	5.43	2.18–13.49	**<0.001**	Yes *(Unadjusted relationship is also positive and significant)*

* Adjusted for sex, age, and scanning level. ^1^ One-tailed *p*-values are reported here. Significant *p*-values for adjusted positive relationships are bolded for emphasis.^2^ Interaction not tested due to insufficient observations.

**Table 7:** Odds ratios matrix for newly created subcategories and distinct behaviours (*n* = 1183 rhesus macaques). Odds ratios are adjusted for sex, age, and scanning level. For unadjusted odds ratios (which omit controls for sex, age and scanning level), see Table A4. Ranges are shown since each behaviour or subcategory was run as both the independent and dependent variable in the models.

Behaviour/Subcategory	1	2	3	4	5	6	7	8	9	10	11	12	13
1. Self-bite/self-hit/ self-injurious behaviour	----	**6.36**– 6.37 *	4.32–4.50 ^1^*	0.62– 0.66 ^1^	0.42– 0.46	0.77– 0.85	0.23– 0.49 ^3a,3c^	0.94– 0.95 ^1^	1.40– 1.41 ^1^	1.47– 1.59 ^1^	0.61– 0.63 ^1^	**6.11**– 7.11 ^4^*	0.44 ^2^
2. Floating limb/leg-lift	----	----	3.16–3.23	0.77– 0.78 ^1^	0.25– 0.26	0.58– 0.59 ^1^	0.09 ^3a^	1.25– 1.29 ^1^	4.26–4.72	0.51– 1.21	0.59– 0.61^1^	**8.31**– 9.52 ^3a^*	2.00 ^2^
3. Self-clasp	----	----	----	1.04– 1.08 ^1^	0.19	0.47– 0.48 ^1^	0.11– 0.12 ^3a^	1.02– 1.24 ^1^	2.66	1.58– 2.55	0.33– 0.40	0.39– 0.40 ^1^	1.39– 1.41^1^
4. Twirl	----	----	----	----	**3.43**– 3.74 ^3b,3c^*	2.25 ^1^*	**4.50**– 4.80 ^1^*	0.97– 1.29	0.62– 0.71^1^	0.44– 0.52	0.15– 0.18 ^1^	0.41– 0.43 ^1^	1.17 ^2^
5. Bounce/rock/swing	----	----	----	----	----	**2.07**– 2.10*	0.29– 0.58 ^3a,3b^	0.47– 0.54	0.39	0.11– 0.15 ^3a^	0.08– 0.09	0.33– 0.35 ^1^	2.06– 2.20 ^2^
6. Hang	----	----	----	----	----	----	0.27– 0.30 ^3a^	1.08– 1.19	0.25– 0.28 ^1^	0.47– 0.62 ^1^	0.10– 0.12 ^1^	0.38– 0.42 ^1^	0.84 ^2^
7. Pace/head-twist	----	----	----	----	----	----	----	0.30– 0.37 ^3a^	0.75– 0.80 ^1^	0.11– 0.12 ^3a^	0.02	0.46– 0.47 ^1^	0.19– 0.26 ^1^
8. Flip	----	----	----	----	----	----	----	----	**2.26**– 2.53 ^1^*	0.21– 0.24 ^3a^	1.11– 1.13 ^1^	0.76– 0.77	0.55 ^2^
9. Eye-poke	----	----	----	----	----	----	----	----	----	1.19– 2.24	0.19 ^1^	2.01– 2.07 ^1^	1.64 ^2^
10. Self-suck	----	----	----	----	----	----	----	----	----	----	0.42– 0.59 ^1^	0.72– 0.85 ^1^	0.44 ^2^
11. Hair-pluck	----	----	----	----	----	----	----	----	----	----	----	0.85– 0.90 ^1^	1.12– 1.22 ^1^
12. Threat-bite	----	----	----	----	----	----	----	----	----	----	----	----	2.50 ^2^
13. Withdrawn	----	----	----	----	----	----	----	----	----	----	----	----	----

* *p* ≤ 0.05. Significant and positive relationships are bolded for emphasis. ^1^ Interaction not tested due to insufficient observations.^2^ Based on a univariable analysis since there were too few observations to fit a multivariable model. ^3^ Only significant contrasts are reported (presence vs. absence in each scanning level): ^3a^ = Rarely scanned, ^3b^ = Moderately scanned,^3c^ = Frequently scanned. ^4^ Threat-bite positively predicted ‘self-bite/self-hit/self-injurious behaviour’ (*OR* = 7.11, *p* = 0.049), but this association was not significant when ‘self-bite/self-hit/self-injurious behaviour’ was the dependent variable (*OR* = 6.11, *p* = 0.064). --- denotes no odds ratio computed.

**Table 8:** Steps involved in creating valid subcategories of abnormal behaviours in laboratory rhesus macaques.

Step 1—Creating Initial Subcategories (Based on Table 3, Table 4, Table 5 and Table 6 and Table A1, Table A2 and Table A3)	Step 2—Expanding Subcategories (Based on Table 7)
Self-biting, self-hitting, and self-injurious behaviours;	1. ‘Self-biting, self-hitting, self-injurious behaviour, floating limb, leg-lift, self-clasping’ subcategory
Floating limb and leg-lift;	
Self-clasping;	
Bouncing, rocking, and swinging;	2. ‘Bouncing, rocking, swinging, twirling and hanging’ subcategory
Twirling;	
Hanging;	
Pacing and head-twisting;	3. ‘Pacing and head-twisting’ subcategory
Flipping;	4. ‘Flipping and eye-poking’ subcategory
Eye-poking;	
Self-sucking;	5. ‘Self-sucking’
Hair-plucking;	6. ‘Hair-plucking’
Threat-biting;	7. ‘Threat-biting’
Withdrawn;	8. ‘Withdrawn’

## Data Availability

The data presented in this study are available on request from the corresponding author.

## Appendix A

**Table A1:** Logistic regression models for bouncing presence (‘motor’ stereotypic behaviour subcategory) ^1^.

Independent Variables	Unadjusted Odds Ratio and *p*-Value	Adjusted Odds Ratio *	95% CI *	*p*-Value *	Positively and Significantly Predicts Bouncing? * *(Result the Same if Age, Sex and Scanning Level not Controlled for?)*
Flipping presence vs. absence	1.29 (0.219)	0.23	0.03–1.80	0.885	No *(Unadjusted relationship is also non-significant, yet positive)*
Twirling presence vs. absence	2.37 (0.013)	0.64	0.08–5.20	0.662	No *(Unadjusted relationship is positive and significant)*
Swinging presence vs. absence	3.08 (<0.001)	2.60	0.99–6.82	0.026	Yes *(Unadjusted relationship is also positive and significant)*
Rocking presence vs. absence	2.78 (<0.001)	2.60	0.90–7.50	0.039	Yes *(Unadjusted relationship is also positive and significant)*

* Adjusted for sex, age, and scanning level. ^1^ One-tailed *p*-values are reported here. Significant *p*-values for adjusted positive relationships are bolded for emphasis.

**Table A2:** Logistic regression models for hair-plucking presence (‘self-stimulation’ subcategory).

Independent Variables	Unadjusted Odds Ratio and *p*-Value	Adjusted Odds Ratio *	95% CI *	*p*-Value *	Positively and Significantly Predicts Hair-Plucking? * *(Result the Same if Age, Sex and Scanning Level not Controlled for?)*
Self-clasping presence vs. absence	0.48 (0.835)	0.33	0.04–2.63	0.835	No *(Unadjusted relationship is also negative)*
Eye-poking presence vs. absence ^2^	0.27 (0.912)	0.19	0.03–1.45	0.946	No *(Unadjusted relationship is also negative)*

* Adjusted for sex, age, and scanning level. ^1^ One-tailed *p*-values are reported here. ^2^ Interaction not tested due to insufficient observations.

**Table A3:** Logistic regression models for floating limb presence (‘postural’ subcategory).

Independent Variables	Unadjusted Odds Ratio and *p*-Value	Adjusted Odds Ratio *	95% CI *	*p*-Value *	Positively and Significantly Predicts Floating Limb? * *(Result the Same if Age, Sex and Scanning Level not Controlled for?)*
Leg-lifting presence vs. absence	10.51 (<0.001)	8.45	0.88–80.78	**0.032**	Yes *(Unadjusted relationship is also positive and significant)*
Withdrawn presence vs. absence ^2^	3.83 (0.073)	2.14	0.42–10.95	0.182	No *(Unadjusted relationship is also positive and non-significant)*

* Adjusted for sex, age, and scanning level. ^1^ One-tailed *p*-values are reported here. Significant *p*-values for adjusted positive relationships are bolded for emphasis. ^2^ Based on a univariable analysis since there were too few observations to fit a multivariable model.

**Table A4:** Unadjusted odds ratios matrix for newly created subcategories and distinct behaviours (*n* = 1183 rhesus macaques).

Behaviour/Subcategory	1	2	3	4	5	6	7	8	9	10	11	12	13
1. Self-bite/self-hit/ self-injurious behaviour	----	10.39 *	4.47 *	1.01	1.04	1.26	0.24– 0.28 ^2a,c^	1.35	2.02 ^3,^*	1.57 ^3,^*	0.58	2.57 *	0.44
2. Floating limb/leg-lift	----	----	6.24 ^3,^*	1.15	0.95	0.66	0.09 ^2a^	1.72 ^3,^*	2.70 ^3,^*	1.72 ^3,^*	0.61	16.67 ^2a,^*	2.00
3. Self-clasp	----	----	----	1.26	0.84	0.50	0.12 ^2a^	1.43	1.40	2.52 ^3,^*	0.41	0.48	1.42
4. Twirl	----	----	----	----	3.63– 4.14 ^2b,c,^*	2.68 *	6.01 *	3.88 ^3,^*	0.84	1.00	0.12	0.62	1.17
5. Bounce/rock/swing	----	----	----	----	----	3.36 *	0.30– 0.58 ^2a,b^	1.18	0.48	0.15 ^2a^	0.30	0.45	1.93
6. Hang	----	----	----	----	----	----	0.28 ^2a^	2.10 ^3,^*	0.29	1.00	0.09	0.44	0.84
7. Pace/head-twist	----	----	----	----	----	----	----	0.32 ^2a^	1.30	0.02 ^2a^	0.18	0.99	0.28
8. Flip	----	----	----	----	----	----	----	----	2.54 *	0.69 ^2a^	0.30	0.94	0.55
9. Eye-poke	----	----	----	----	----	----	----	----	----	1.48	0.17	2.94 ^3,^*	1.64
10. Self-suck	----	----	----	----	----	----	----	----	----	----	0.09 ^3,^*	0.74	0.44
11. Hair-pluck	----	----	----	----	----	----	----	----	----	----	----	0.87	2.98
12. Threat-bite	----	----	----	----	----	----	----	----	----	----	----	----	2.50
13. Withdrawn	----	----	----	----	----	----	----	----	----	----	----	----	----

* *p* ≤ 0.05. ^1^ One-tailed *p*-values are reported here. ^2 a,b,c^ Univariable models run among each group of scanning: ^a^ = Rarely scanned, ^b^ = Moderately scanned, ^c^ = Frequently scanned. ^3^ Relationship becomes non-significant (*p* > 0.05) after adjusting for sex, age, and scanning level (see Table 7). --- denotes no odds ratio computed.
